# In acid-aminopyrimidine continuum: experimental and computational studies of furan tetracarboxylate-2-aminopyrimidinium salt†

**DOI:** 10.1039/d1ra01714d

**Published:** 2021-06-17

**Authors:** Utsav Garg, Yasser Azim, Mahboob Alam

**Affiliations:** Department of Applied Chemistry, Zakir Husain College of Engineering & Technology, Aligarh Muslim University Aligarh 202002 Uttar Pradesh India yasser.azim@gmail.com; Division of Chemistry & Biotechnology, Dongguk University 123 Dongdae-ro Gyeongju Republic of Korea mahboobchem@gmail.com

## Abstract

Salts and cocrystals are the two important solid forms when a carboxylic acid crystallizes with an aminopyrimidine base such that the extent of proton transfer distinguishes between them. The Δp*K*_a_ value (p*K*_a_(base) − p*K*_a_(acid)) predicts whether the proton transfer will occur or not. However, the Δp*K*_a_ range, 0 < Δp*K*_a_ < 3, is elusive where the formation of cocrystal or salt cannot be predicted. The current study has been done to obtain a generalization in this elusive range with the Cambridge Structural Database (CSD). Based on the generalization, a novel salt (FTCA)^−^(2-AP)^+^ of furantetracarboxylic acid (FTCA) with 2-aminopyrimidine (2-AP) is obtained. The structural confirmation was done by single-crystal X-ray diffraction (SCXRD). Density functional theory (DFT) calculations were performed at the IEF-PCM-B3LYP-D3/6-311G(d,p) level to optimize the geometrical coordinates of salt for frontier molecular orbitals (FMOs) and molecular electrostatic potential (MESP). The geometrical parameters of most of the atoms of the optimized salt structure were comparable with SCXRD data. Additionally, results of other computational methods such as *ab initio* (Hartree–Fock; HF and second-order-Møller–Plesset perturbation; MP2) and semi-empirical were also compared with experimental results of the salt. Quantum theory of atoms in molecules (QTAIM), reduced density gradient (RDG), and natural bond orbital (NBO) analyses were done to calculate the strength and nature of non-covalent interactions present in the salt. Furthermore, Hirshfeld surface analysis, interaction energy calculations, and total energy frameworks were performed for qualitative and quantitative estimations of strong and weak intermolecular interactions.

## Abstract 2

Generalization in the elusive Δp*K*_a_ range, experimental and computational studies of furan tetracarboxylate-2-aminopyrimidinium salt.

## Graphical abstract

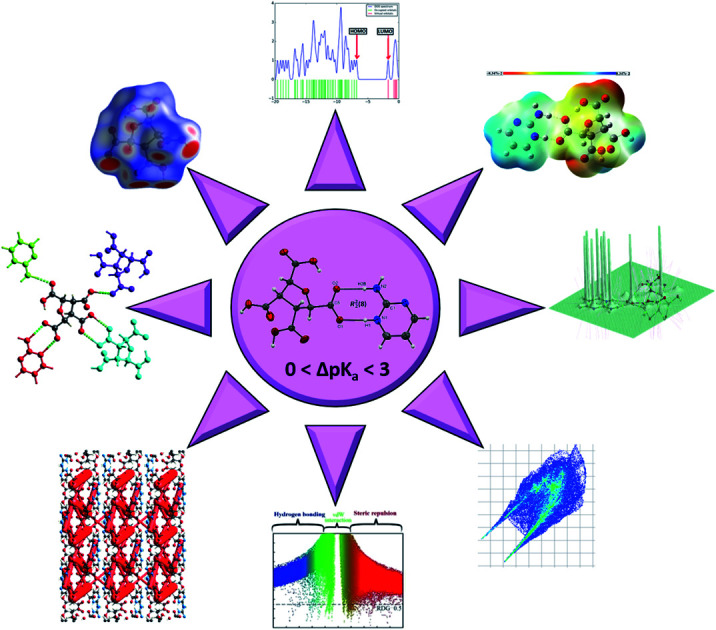

## 1. Introduction

Crystal engineering (CE) is an emerging research area encompassing various domains of physics, chemistry, biology, engineering, materials science, and pharmaceuticals.^1^ The three aspects of CE *viz.* understanding intermolecular interactions, designing new solids, and desired physical and chemical properties have been explored extensively in the past ten years.^2^ In CE's infancy stage, the work started with developing new crystals and their deposition in CSD. The storing and retrieving of crystallographic data in CSD worked as a driving force in understanding intermolecular interactions in terms of robustness of supramolecular synthons that led to the design of new solids having opulent importance both in academia and industry.^3,4^ Molecular simulation by computer has now evolved into a robust companion for the synthetic effort and the availability of a database of millions of crystal structures to test new theories and algorithms.^5^ The advancement of computational studies (like DFT calculations, FMOs, QTAIM, RDG, NBO, Hirshfeld surfaces, *etc.*) for understanding and utilization of non-covalent interactions (such as hydrogen bond, halogen bond, van der waal interactions, π⋯π interactions, electrostatic interactions, *etc.*) has proved its potential from rational design to predicted properties and applications in CE.^5,6^

Pyrimidine and aminopyrimidine bases are the essential components of different nucleic acids (including DNA/RNA) that interact through hydrogen bonding for genetic information transfer.^7–9^ Likewise, many biologically important compounds, namely drugs, nucleic acids, plant hormones, *etc.*, consist of carboxyl groups in their chain.^10–13^ It is well known that the carboxyl group of proteins interacts with pyrimidine moiety of nucleic acids causing protein–nucleic acid recognition.^14–18^ This recognition has been extensively utilized to design salts and cocrystals for Active Pharmaceutical Ingredients (API) and model compounds. In literature, two robust synthons, Linear HeteroTetramer (LHT) and HeteroTrimer (HT),^19,20^ are generally found in salt/cocrystal of aminopyrimidine and carboxylic acid.^21–25^ The distinction between salt and cocrystal is an important aspect from a regulatory and legal perspective. The extent of proton transfer only differentiates salts and cocrystals; however, both solid forms have shown potent advantages in improving physicochemical and pharmacological properties.^26–29^ After so many years of development of cocrystals, its definition is still in debate considering it a new entity or not.^30^ Even the EMA (European Medical Agency) regulations are distinct from the USFDA (United States Food and Drug Administration) regulations for cocrystal approval.^26^ In this context, a salt maybe better than a cocrystal as the compendial guidelines for salt approval are same by both regulatory bodies.^31^ The prediction of getting a cocrystal or salt can be made with Δp*K*_a_ value, described by the equation, Δp*K*_a_ = p*K*_a_(base) − p*K*_a_(acid). (i) For Δp*K*_a_ > 3, higher the chance of salt formation (also called charge transfer complex), (ii) for Δp*K*_a_ < 0, more likely to be cocrystal formation, (iii) for 0 < Δp*K*_a_ < 3, the predictability is elusive as may be salt or cocrystal formation takes place.^32^

**Scheme 1:**
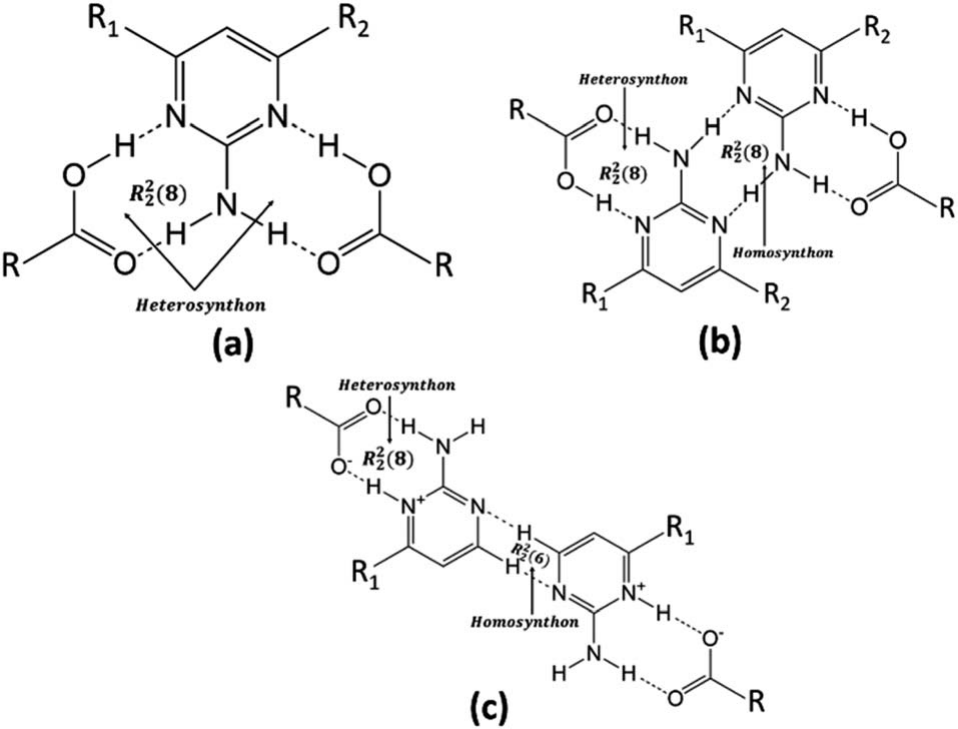
General structures of (a) HT synthon (b) popular LHT synthon *via* N–H⋯N interaction (c) proton transferred LHT synthon (CH-LHT) *via* C–H⋯N interaction found in present case.

The authors of the same lab tried to address the robustness of LHT over HT synthon and predictability of the formation of a salt/cocrystal based on Δp*K*_a_ (Scheme 1). However, the work was restricted to a number of acid groups (an extended homolog/extended conjugation) and a number of acceptor and donor groups on the pyrimidine moiety. The plausibility for the robustness of LHT over HT synthon was reasoned, but the elusive Δp*K*_a_ range (0 < Δp*K*_a_ < 3) was still ambiguous.^33,34^ In continuum to the previous work and to resolve the ambiguity of Δp*K*_a_ for a generalization, different computational studies and CSD data have been extensively utilized in the current study. The predicted novel salt has been obtained experimentally from FTCA and 2-AP, whose Δp*K*_a_ belongs to the elusive range.

## 2. Experimental

### 2.1. Materials

All chemicals (reagent quality) were obtained from commercial sources and used without further purification. 2-Aminopyrimidine (2-AP, purity >99%) was purchased from Sigma Aldrich, Germany. Furan tetracarboxylic acid (FTCA, purity >98%) and solvents of analytical grade were obtained from Fisher Scientific (India Limited, Mumbai).

### 2.2. Crystallographic database (CSD)

To date, 123 different crystal structures encompassing carboxylic acid-aminopyrimidine synthon have been reported and are available in CSD.^35^ Seventy of them (approx. 56.9%) are obtained as LHT, forty-two (approx. 34.1%) as HT, and the remaining 9% as a different synthon. The different pairs of acid-aminopyrimidine synthon are found as salt or cocrystal. However, no salt/cocrystal of FTCA with any coformer is yet reported in CSD. Also, no salt/cocrystal is reported for any tetracarboxylic acid with 2-AP or its derivative. Thus, it is the first attempt to report supramolecular synthon for a tetracarboxylic acid (*i.e.*, FTCA) with 2-AP. Conquest program (version 1) was employed for searching deposited structures.^35^

### 2.3. Preparation of (FTCA)^−^(2-AP)^+^ salt

(FTCA)^−^(2-AP)^+^ salt was prepared by solvent evaporation method. A 1 : 1 mixture of FTCA (99.26 mg, 0.4 mmol) and 2-AP (72.08 mg. 0.4 mmol) were dissolved in 8 mL (approx.) ethanol (C_2_H_5_OH) to make a clear solution. The resulting solution was filtered, and the filtrate was left to slowly evaporate at room temperature. The good quality single crystals (colourless block) suitable for X-ray diffraction studies were obtained after a week.

### 2.4. p*K*_a_ calculations

ChemAxon software was used to calculate p*K*_a_ values for different carboxylic acids and 2-AP.^36^

### 2.5. Single crystal X-ray diffraction (SCXRD)

High resolution single crystal X-ray measurements for (FTCA)^−^(2-AP)^+^ salt were carried out at 293 K on the SuperNova diffractometer (UK), with the dual-source (Mo and Cu) and Eos CCD detector, using Cu-Kα radiation (1.54184 Å). Data acquisition, reduction and absorption correction was done by using CrysAlisPRO program.^37^ The structure was solved in Olex2 (v.1.3)^38,39^ suite of programs by direct methods using ShelXS^40^ and refined by the full matrix on F2 using ShelXL.^41^ All non-hydrogen atoms were refined anisotropically, and after that, all H-atoms attached to non-C atoms have been located from the difference Fourier map and rest H-atoms were refined as riding atoms using isotropic displacement parameters. Diagrams and publication material were generated using PLATON^42^ and MERCURY (v.2020.2.0)^43^ software.

### 2.6. Computational studies

DFT calculations were performed using Gaussian 09 (ref. 44) through the Web MO interface.^45^ The hybrid functional, Becke three parameters exchange functional, the Lee–Yang–Par (LYP) correlation functional, including Grimme's D3 correction for non-covalent interactions, was used in the present theoretical calculations. Initial geometry retrieved from asymmetric unit of the crystal structure was subjected to perform a single point energy and geometry optimization followed by frequency calculation to check whether the structure corresponds to a stationary point or not at the IEF-PCM-B3LYP-D3/6-311G(d,p) level of theory^46–48^ in the solution phase (ethanol). The integrated integral equation formalism for the polarizable continuum model (IEF-PCM) was used to stabilize salt form otherwise the studied molecule would be returned to neutral form in the gas phase. Some calculations were also carried out in gas phase to compare descriptors of the salt and its constituents. No imaginary values of frequency were found indicated that the optimized geometry at the potential energy surface was minimal. The molecular geometry of salt was also optimized by other computational methods like *ab initio* (IEF-PCM-MP2/6-311G(d,p) and IEF-PCM-HF/6-311G(d,p)) and semi-empirical (IEF-PCM-PM6) in the solution phase (ethanol) to compare some important geometrical parameters obtained from experimental data. Furthermore, DFT calculations were done on a hypothetical cocrystal structure using the same basis set as in salt optimization to calculate and compare the binding energy (B.E.) of salt and cocrystal.

The FMOs and MESP mapping plots of the (FTCA)^−^(2-AP)^+^ and its fragments were generated using a GaussView 5 visualization program.^49^ Pictorial presentation and topological properties at bond critical points were explained within the framework of QTAIM using the AIMALL package. The strength of long-range forces was demonstrated through the Multiwfn program^50^ and the isosurface visualization is obtained by the VMD program.^51^ For H-bonds interactions, the NBO analysis was carried out by the NBO code^52^ as implemented in Gaussian 09.

Crystal Explorer 17 program was used to generate different molecular Hirshfeld surfaces, 2-D fingerprint plots,^53,54^ interaction energy, and total energy frameworks^55^ for better understanding the nature of intermolecular interactions present in the crystal structure. A crystallographic information file (CIF) of (FTCA)^−^(2-AP)^+^ salt was used as input for the analysis. The Hirshfeld surfaces were mapped with different properties, namely, *d*_norm_,^56^ curvedness,^57^ shape-index,^57^ and electrostatic potential.^54^ The bond lengths of hydrogen atoms involved in interactions were normalized to standard neutron values (as available in Crystal Explorer17) to generate 2-D fingerprint plots. The interaction energies were calculated using a dispersion-corrected CE-B3LYP/6-31G(d,p) quantum level of theory available in Crystal Explorer17. The total intermolecular energy is the sum of energies of four main components, comprising electrostatic, dispersion, polarization, and exchange-repulsion with scale factors of 1.057, 0.871, 0.740, and 0.618, respectively. The graphical representation of individual energy components *viz. E*_elec_, *E*_dis_, and *E*_tot_ (also called simulation of energy frameworks) were done and depicted as colour-coded cylinders joining the centroids of interacting molecular pairs.

## 3. Results and discussions

The reported salts/cocrystals of derivatives of aromatic acids with 2-AP were analysed to increase the predictability of getting cocrystal or salt in the elusive Δp*K*_a_ range (*i.e.*, 0 < Δp*K*_a_ < 3). Table 1 lists supramolecular synthon, and Δp*K*_a_ values of some CSD reported salts/cocrystals of carboxylic acids with 2-AP. It was observed that LHT was dominant over HT synthon in both cocrystal and salt forms; however, there were few cases also in which neither LHT nor HT was formed. The cocrystal formation occurred in all the cases where Δp*K*_a_ was negative, whereas no case was found for Δp*K*_a_ greater than 3. A significant observation was ascertained in the elusive Δp*K*_a_ range. The unsubstituted aromatic acids like benzoic acid formed cocrystal with 2-AP (as Δp*K*_a_ < 0), whereas the monosubstituted aromatic acids formed either cocrystal or salt with 2-AP. On further increasing the substitution of groups showing negative inductive effect (−*I* effect) on aromatic ring, the predictability of getting the salt rose to almost 100%. Furan tetracarboxylic acid (FTCA), a five-membered heterocyclic aromatic ring containing four carboxyl groups, was chosen to verify this generalization. FTCA or tetrahydrofuran-2,3,4,5-tetracarboxylic acid (IUPAC – oxolane-2,3,4,5-tetracarboxylic acid) is a strong acid, and it forms a series of well-defined mono salts, which are stable to hydrochloric acid, with a variety of bases.^58^ Furan-based substituted compounds showed up-and-coming biological applications such as anti-cancer, antimicrobial, anti-hyperglycemic, and analgesic.^59^ The Δp*K*_a_ value of FTCA (p*K*_a_ = 2.38) with 2-AP (p*K*_a_ = 3.62) is 1.24, and it lies in the elusive Δp*K*_a_ range. The generalization predicted the proton transfer between FTCA and 2-AP due to the presence of more than two –I effect groups on the aromatic tetrahydrofuran ring. The single-crystal structure showed our prediction to be correct as a salt formation occurred with FTCA as anion and 2-AP as cation. All the crystallographic data and experimental details for the (FTCA)^−^(2-AP)^+^ salt are depicted in Table S1,† while Table S2† represents some important hydrogen-bond parameters for the prepared salt. Different computational studies were done to find the plausible reason behind the occurrence of salt and for a better understanding of the nature of intermolecular interactions (including O–H⋯O, N–H⋯O and C–H⋯N) present in the crystal structure of (FTCA)^−^(2-AP)^+^ salt.

**Table 1:** Δp*K*_a_ of reported salt/cocrystal of different carboxylic acids with 2-AP

Base (p*K*_a_)	Acid (p*K*_a_)	Δp*K*_a_	Synthon	Salt/cocrystal	CSD refcode
2-AP (3.62)	Cocrystal range (Δp*K*_a_ < 0)				
	4-Aminobenzoic acid (4.77)	−1.15	LHT	Cocrystal	LEWPUY
	Indole-3-acetic acid (4.66)	−1.04	LHT	Cocrystal	JIQCAN
	Ibuprofen (4.43)	−0.81	LHT	Cocrystal	TAWSOB
	3,3,3-Triphenylpropanoic acid (4.25)	−0.63	LHT	Cocrystal	GIMPAU
	1-Naphthalene acetic acid (4.23)	−0.61	LHT	Cocrystal	YUKVIM
	*N*-Methylpyrrole-2-carboxylic acid (4.11)	−0.49	LHT	Cocrystal	JIQCER
	Benzoic acid (4.20)	−0.58	LHT	Cocrystal	NUKWOG
	4-Chlorobenzoic acid (3.98)	−0.36	LHT	Cocrystal	MOZBAG
	Camphoric acid (4.07)	−0.45	HT	Cocrystal	KIXVES
	*p*-Phenylenediacetic acid (4.03)	−0.41	HT	Cocrystal	GODQIZ
	3-Bromo benzoic acid (3.93)	−0.31	HT	Cocrystal	MOZBOU
	Glutaric acid (3.76)	−0.14	HT	Cocrystal	JOYJAK
	Succinic acid (3.55)	−0.07	HT	Cocrystal	SERMOR
	Elusive range (0 < Δp*K*_a_ < 3)				
	3-Aminobenzoic acid (3.07)	0.55	LHT	Cocrystal	ZAJJEZ
	2-Bromobenzoic acid (2.85)	0.77	LHT	Cocrystal	MOZBEK
	Terephthalic acid (3.32)	0.30	HT	Cocrystal	SUVJEY
	*o*-Phthalic acid (2.94)	0.68	HT	Cocrystal	ZAJHOH
	Naphthalene-1,4-dicarboxylic acid (2.71)	0.91	HT	Cocrystal	OFUHUS
	4,5-Dichloro phthalic acid (2.35)	1.27	LHT	Salt	LORWEV
	2-Nitrobenzoic acid (2.16)	1.46	LHT	Salt	CIHYEY
	2,4,6-Trinitrobenzoic acid (0.65)	2.97	LHT	Salt	NUTRAW
	Pentafluorobenzoic acid (1.48)	2.14	Neither LHT nor HT	Salt	MOYZOR
	3,5-Dinitrosalicylic acid (1.31)	2.31	Neither LHT nor HT	Salt	AJECIB
	2,6-Dihydroxybenzoic acid (1.64)	1.98	Neither LHT nor HT	Salt	LEWPOS
	Salicylic acid (2.79)	0.83	Neither LHT nor HT	Salt	LEWROU

### 3.1. Molecular and supramolecular structure of (1 : 1) furantetracarboxylic acid:2-aminopyrimidine ((FTCA)^−^(2-AP)^+^) salt

**Fig. 1:**
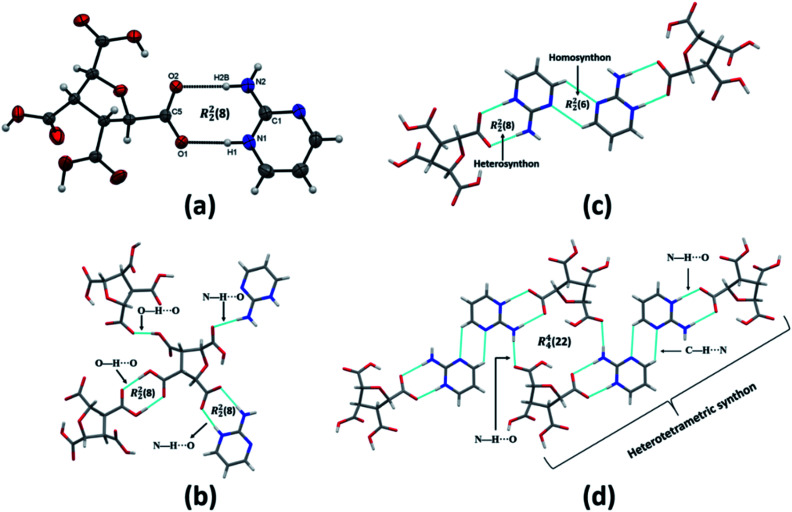
(a) ORTEP representation of (FTCA)^−^(2-AP)^+^ salt asymmetric unit (thermal ellipsoids were drawn at 50% probability level); (b) a view of four different synthons surrounding the FTCA molecule; (c) LHT synthon; (d) a view of crystal lattice composed of four-component centrosymmetric motif, R^4^_4_(22), connected by N–H⋯O hydrogen bonds.

(FTCA)^−^(2-AP)^+^ crystallizes in monoclinic, *P*2_1_/*c* space group in the asymmetric unit (ASU) with FTCA as anion and 2-AP as cation (Fig. 1(a)). The bond lengths of C–O (1.253(3) Å) and C=O (1.250(3) Å) of the carboxylic acid group are close enough to each other, confirming that the protonation takes place. The FTCA and 2-AP molecules in the ASU are connected through two N–H⋯O (*d* = 1.860 Å and *d* = 1.994 Å) hydrogen bonds to form R^2^_2_(8) motif through graph-set descriptor.^60^ FTCA consists of four carboxylic acid groups, and each carboxylic acid interacts differently in a salt. The two carboxylic acid groups of FTCA interact with two 2-AP molecules *via* N–H⋯O interaction; however, one form one-point synthon and the other form two-point synthon. The rest two carboxylic acid groups of FTCA form homodimer with carboxylic acid groups of other FTCA molecules. Herein, one form one-point synthon and another form two-point synthon *via* O–H⋯O interactions (Fig. 1(b)). On expanding the crystal structure, a linear heterotetramer (CH-LHT) with alternate homo and hetero synthon is formed between aminopyrimidine and acid group (*via* N–H⋯O & O–H⋯N) and between aminopyrimidine groups (*via* C–H⋯N and C–H⋯N) respectively. Earlier, the authors of the same lab have seen the robustness of LHT over HT synthon. When an LHT synthon occurs, a R^2^_2_(8) heterodimer is formed between aminopyrimidine and acid group (*via* N–H⋯O & O–H⋯N) and a R^2^_2_(8) homodimer is formed between aminopyrimidine groups (*via* N–H⋯N and N–H⋯N), depicted in Scheme 1.^33,34^ In the present case, like general LHT synthon, a R^2^_2_(8) heterodimer is formed between aminopyrimidine and acid group (*via* N–H⋯O & O–H⋯N); however, in place of R^2^_2_(8) homodimer, a rare R^2^_2_(6) homodimer is formed between two aminopyrimidine groups (*via* C–H⋯N and C–H⋯N). The dihedral angle between the mean planes of O1–C5–O2 of FTCA and N1–C1–N2 of 2-AP is 23.50°, whereas the dihedral angle between the mean planes of N2–C1–N3 of one 2-AP and N2–C1–N3 of another 2-AP is 0.00° (Fig. 1(c)). In the crystal lattice, a four-component centrosymmetric motif, R^4^_4_(22), composed of two (FTCA)^−^ and two (AP)^+^ molecules, is also observed. Here, the two heterotetramers are connected by strong N–H⋯O forces (Fig. 1(d)).

### 3.2. Optimized structure, FMOs, and MESP

**Fig. 2:**
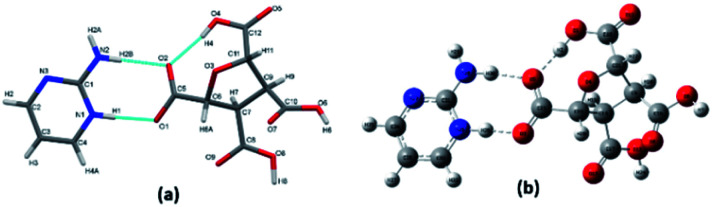
(a) Asymmetric unit of (FTCA)^−^(2-AP)^+^ salt. (b) Optimized structure of (FTCA)^−^(2-AP)^+^ with the labelling of atoms.

The optimized structure of the (FTCA)^−^(2-AP)^+^ salt was obtained by applying (DFT/B3LYP-D3) method with the 6-311G(d,p) basis set (Fig. 2) in the solution phase.

**Scheme 2:**
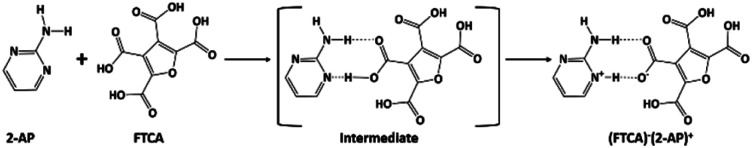
Illustration of the formation of (FTCA)^−^(2-AP)^+^ salt from its constituents *via* intermediate formation.

The parameters such as bond lengths, bond angles, and dihedral angles of (FTCA)^−^(2-AP)^+^ salt are listed in Table S3† and compared with selected parameters obtained from salt's experimental data. The calculated minimum molecular energy and dipole moment of the optimized salt structure and its constituents are shown in Tables 2 and S4† in the solution and gas phases, respectively, although these values vary depending on the method used for the optimization process. Salt optimized in the solution phase was 17.267 kcal mol^−1^ more stable compared to the structure optimized in the gas phase. The calculated bond length of N^+^25–H26 connected to O^−^3 of FTCA through hydrogen bond indicated the marginal deviation (∼0.213 Å) from experimental SCXRD results. Similarly, a modest difference (∼0.291 Å) from the SCXRD findings was found in the bond lengths of hydrogen bond N^+^25–H26⋯O^−^3—C23 linking FTCA and 2-AP together for salt formation (Scheme 2). Other noticeable deviations in bond lengths were found in case of N28–H29 and N28–H30 by 0.14 and 0.15 Å, respectively. Significant deviation of more than ∼4° was noticed in C23–O3–H26, C23–O5–H30, and H26–H25–C31 involving hydrogen bond formation because values of hydrogen atoms could not be derived precisely from SCXRD experiment.^61^ Furthermore, any other inconsistencies found between the theoretical data and SCXRD could be attributed to the fact that the theoretical calculations were carried out for isolated (FTCA)^−^(2-AP)^+^ in the solution and gaseous phase while the SCXRD data was collected for bulk phase in solid state. The involvement of crystal field and intermolecular attractions in solid state bind crystals resulting in the difference in the optimized parameters between the calculated and experimental values, particularly in the dihedral angles for large deviations.

**Table 2:** Calculated quantum chemical parameters (in eV) of FTCA, 2-AP and (FTCA)^−^(2-AP)^+^ salt using DFT/B3LYP-D3 method in the solution phase

Parameters	FTCA	2-AP	(FTCA)^−^(2-AP)^+^ salt
*E*_LUMO_	−0.4	−1.14	−2.01
*E*_HOMO_	−7.77	−6.37	−7.27
Δ*E*_g_ (*E*_LUMO_ − *E*_HOMO_)	7.37	5.23	5.26
Minimum SCF energy (kcal mol^−1^)	−619 368.66	−200 673.46	−820 087.24
Dipole moment (Debye)	1.74	0.06	11.72
Ionization potential (*I*)	7.77	6.37	7.27
Electron affinity (*A*)	0.4	1.14	2.01
Chemical hardness (*η*)	3.685	2.615	2.63
Chemical softness (*S*)	0.136	0.191	0.190
Electronegativity (*χ*)	4.085	3.755	4.64
Electronic chemical potential (*μ*)	−4.085	−3.755	−4.64
Electrophilicity index (*ω*)	2.269	2.693	4.090

Likewise, other computational approaches such as *ab initio* (IEF-PCM-MP2/6-311G(d,p) and IEF-PCM-HF/6-311G(d,p)) and semi-empirical (IEF-PCM-PM6) method were used in the same way as DFT to optimize the salt structure. The obtained computational results were compared in terms of bond lengths and bond angles with the experimental data (see Table 3) obtained from SCXRD. The optimized salt structure using DFT approach was most stable among other applied computational methods with the highest negative SCF energy, *i.e.*, −820 087.24 kcal mol^−1^. Most geometrical parameters of salt obtained using DFT at the IEF-PCM-B3LYP-D3/6-311G(d,p) level of theory were comparable with SCXRD data, except for bond lengths involved in the formation of hydrogen bonds between FTCA and 2-AP. DFT calculations were also utilized to demonstrate the formation of salt over cocrystal. The binding energies (B.E.) of optimized salt and cocrystal (hypothetical) structure were calculated in solution phase (Fig. S1†) such that the higher B.E. favoured salt formation (B.E. = −188.78 kJ mol^−1^) over cocrystal (B.E. = −68.58 kJ mol^−1^). The complexation is energetically advantageous and spontaneous when the binding energy is negative.

**Table 3:** Comparison of selected experimental structural parameters (bond lengths and bond angles) of (FTCA)^−^(2-AP)^+^ salt with optimized structural parameters derived from different computational studies^a^

Geometric parameters		Computational methods				Experimental values
		Density functional theory (DFT)	*Ab initio* method (HF)	*Ab initio* method (MP2)	Semi-empirical	
Bond length (Å)	N25–H26	1.07	1.02	1.09	1.09	0.86
	N25–C31	1.36	1.34	1.35	1.41	1.34
	C31–N28	1.32	1.31	1.34	1.35	1.32
	N28–H30	1.03	1.00	1.03	1.04	0.86
	O3–C23	1.24	1.22	1.26	1.25	1.25
	C23–O5	1.26	1.24	1.27	1.27	1.25
	O5–H2	1.60	1.70	1.60	1.53	1.81
Angles (°)	H26–N25–C31	119.9	119.8	120.7	120.5	119.5
	N25–C31–N28	118.8	119.1	118.5	119.4	119.4
	C31–N28–H30	121.8	122.0	119.2	122.1	120.0
	H30–O5–C23	118.4	117.9	118.2	123.5	110.0
	O5–C23–O3	125.9	126.2	126.5	123.8	125.9
	C23–C19–O4	109.0	108.8	109.1	107.0	109.2
	N25–C31–N27	121.3	121.2	122.1	121.0	121.6
Minimum SCF energy (kcal mol^−1^)		−820 087.24	−815 492.66	−817 944.06	−377.87	

a Numbering scheme is taken from Fig. 2(b).

Frontier molecular orbitals (FMOs) refer to the highest occupied molecular orbital (HOMO) and the lowest unoccupied molecular orbital (LUMO) that play a significant role in determining the electronic, optical, and chemical properties.^62^ Their energies (HOMO and LUMO) were predicted using the same level theory as that applied to the optimization of (FTCA)^−^(2-AP)^+^ salt in the solution phase and gas phase. The energy difference, also known as energy gap (Δ*E*_g_), between HOMO and LUMO orbitals is considerably answerable for charge transfer, chemical reactivity, and thermodynamic/kinetic stability of molecule. The value of Δ*E*_g_ indicates the possibility of ultimate charge transfer reactions in molecules, acting as soft molecules with lower kinetic stability and high chemical reactivity in the gas phase^63^ and, on the other hand, when salt was optimized in the solvent phase, the value of the energy gap increased with the stabilized hydrogen bond formed between polar atoms (N^+^25–H26⋯O^−^3–C23) leading to salt stability.

**Fig. 3:**
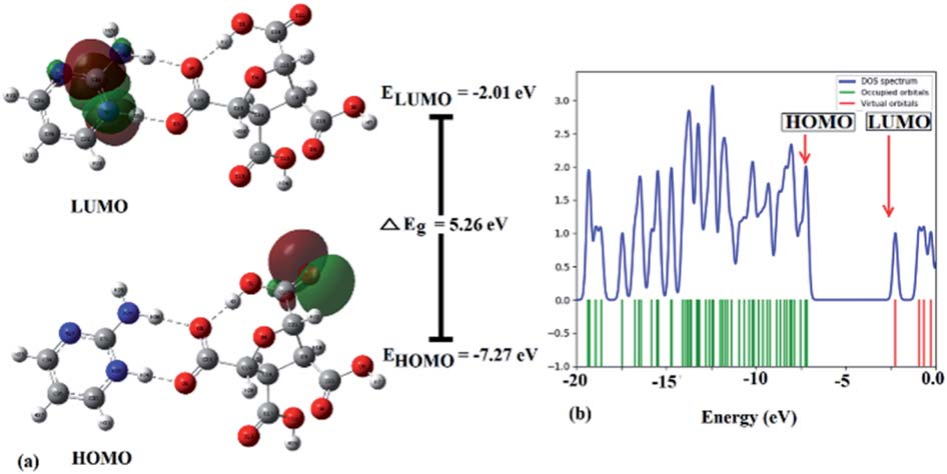
(a) FMOs plot, energies of HOMO & LUMO, energy gap (Δ*E*_g_), and (b) DOS diagram of (FTCA)^−^(2-AP)^+^ salt in solution phase.

The isodensity surface plots of (FTCA)^−^(2-AP)^+^ salt (Fig. S2†) displayed that the HOMO was localized mainly on three carbon atoms of 2-AP ring, whereas the LUMO was localized entirely on nitrogen atoms of 2-AP and H–N–H function in the gas phase while in the solution phase it was found that HOMO was located on carboxylic group of FTCA while LUMO was localized on the N25, C31, N27, and N28 atoms of 2-AP (see Fig. 3(a)). FMOs of the 2-AP and FTCA were also computed using the same level theory as applied to the optimization of (FTCA)^−^(2-AP)^+^ salt, and the pictorial diagram is given in supplementary information (Fig. S3 and S4).† Computed Δ*E*_g_ obtained for (FTCA)^−^(2-AP)^+^ salt were found to be much smaller than its constituents (FTCA and 2-AP) indicated potential charge transfer interactions and high chemical reactivity in salt in the gas phase while in the solution phase, energy gap (HOMO and LUMO) was not found to be much shrinking compared to its salt constitutions showing stability after formation of salt. A variety of quantum mechanical (QM) reactivity descriptors *viz.* chemical hardness (*η*), chemical softness (*S*), electronegativity (*χ*), electronic chemical potential (*μ*), and global electrophilicity index(*ω*) were also calculated with the help of following equations (listed in Table 2).^29^1
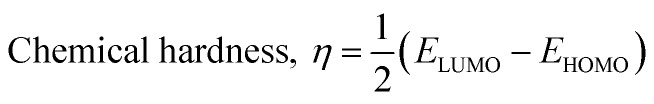
2
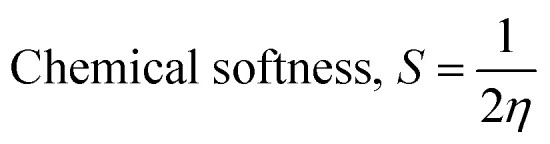
3
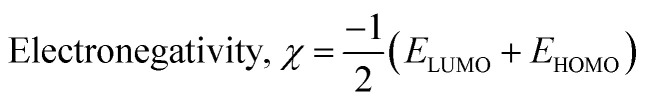
4
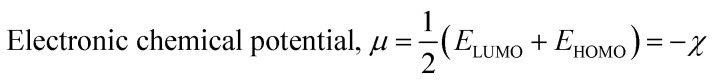
5
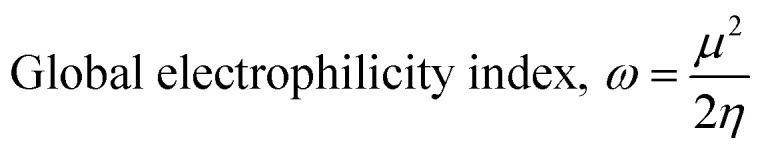


The electronic density of states (DOS) plot, visualized by Gauss-Sum 3.0 Program, shows the density of available states and composition of the molecular orbitals within a particular energy range, such that a high DOS, at a specific energy level, implying that multiple accessible states for occupation are available. Simultaneously, a DOS of zero triggers no states at that particular energy level (Fig. 3(b)). Total DOS also helps to understand occupied molecular orbitals and unoccupied molecular orbitals of the corresponding chemical substances.

**Fig. 4:**
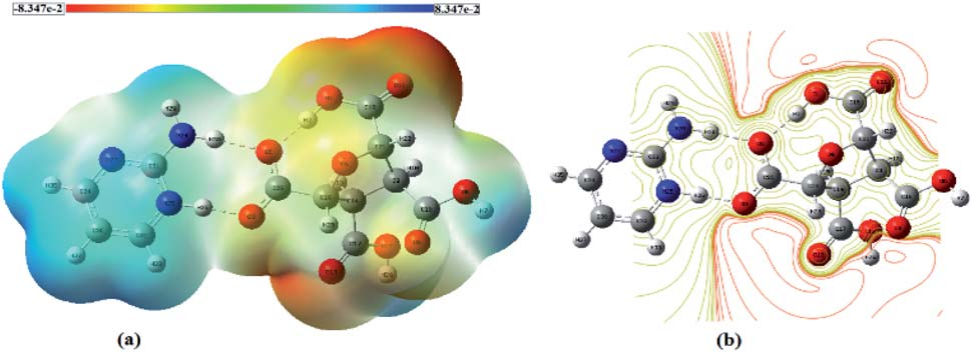
(a) MESP map and (b) 2D contour map of (FTCA)^−^(2-AP)^+^ salt.

The molecular electrostatic potential (MESP) diagram, a plot of electrostatic potential (ESP) mapped on to the constant total electron density (ED) surface, of (FTCA)^−^(2-AP)^+^salt was generated by using B3LYP-D3/6-311G(d,p) method with colour range −8.3 × e^−2^ (deepest red) to +8.3 × e^−2^ (deepest blue) (Fig. 4(a)). The MESP diagrams of 2-AP (Fig. S5(a)†) and FTCA (Fig. S5(b)†) were also plotted to know the reactive sites of individual chemical identities. MESP of 2-AP displayed two favourable binding sites, *i.e.*, negative potential over N1 & N2 atoms and positive potential over –NH_2_ functionality. MESP of FTCA displayed positive potential concentrated over –OH of the carboxyl group that participated in proton transfer from FTCA to 2-AP connected *via* hydrogen bond (Scheme 2). Similarly, the carboxylic group's negative potential over O1 interacted with the positive potential of NH_2_ group through hydrogen bond formation. Analysis of MESP and 2D contour map is quite informative in finding the donor and acceptor regions of the electron and the preferential binding sites. Different colours represent the intensity of ESP on the MESP surface of (FTCA)^−^(2-AP)^+^ salt (Fig. 4(a)); the red reflects the most electronegative potential, blue reflect the most electropositive potential while green coloured areas are for neutral sites. The oxygen atoms O3 and O5 of FTCA are involved in hydrogen bond formation with 2-AP in (FTCA)^−^(2-AP)^+^salt; thereby, these oxygen atoms are not favourable sites for nucleophilic attack. The remaining oxygen atoms have high negative potential and make themselves as a site of nucleophilic attack region. The H29 atom of the 2-AP amine group reveals a positive potential region and contributes to a probable electrophilic attack region with a blue colour surrounding it. Fig. 4(b) displays the contour map of the total ESP of (FTCA)^−^(2-AP) + salt and endorses the various negative and positive potential sites of the molecule as a function of the surface area of the total electron density. The red colour lines signify a highly reactive site, and the yellow colour lines indicate less reactive sites. In the 2D-contour diagram, the dark red lines surrounding some of the oxygen atoms have a negative potential, while the green colour is scattered in a positive potential region depending on the contour representation of the ESP at IEF-PCM-B3LYP-D3/6-311G(d,p) level.

### 3.3. QTAIM topology, RDG, and NBO analysis

**Fig. 5:**
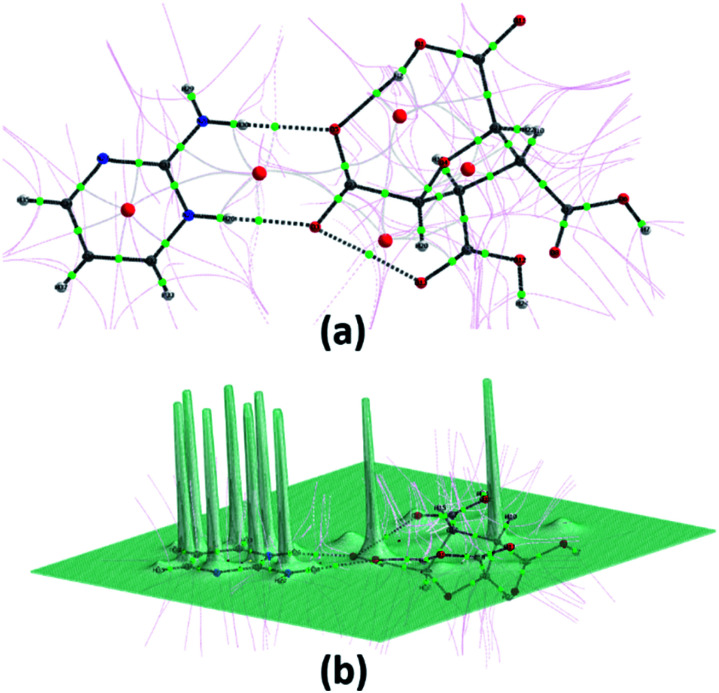
(a) Graphical representation of bond critical points (small green spheres), ring critical points (small red sphere), and bond paths (black lines) using the AIM program. (b) Relief map of the electron density of (FTCA)^−^(2-AP)^+^ salt.

The quantum theory of atoms in a molecule (QTAIM) is used to characterize the covalent and non-covalent interactions present in a molecule such that the strength and nature of hydrogen bonds (intra/inter-molecular) can also be determined.^64^ The interactions between atoms are analyzed by using different topological characteristics *viz.* Electron density (*ρ*_(*r*)_), Laplacian density (∇^2^*ρ*_(*r*)_), and total energy density (*H*) including some other parameters such as ellipticity (*ε*), kinetic energy density (*G*) and potential energy density (*V*) at Bond Critical Points (BCPs) of interacting atoms or fragments (Fig. 5(a) and (b)). These parameters work as a standard for hydrogen bond as suggested by Koch and Popelier: (i) for *H* < 0 and ∇^2^*ρ*_(*r*)_ < 0, strong hydrogen bond will occur and of covalent nature; (ii) for *H* > 0 and ∇^2^*ρ*_(*r*)_ < 0, medium hydrogen bond will occur and of partially covalent nature, and (iii) for *H* > 0 and ∇^2^*ρ*_(*r*)_ > 0, weak hydrogen bond will occur and of electrostatic nature.^65^ QTAIM topology has been used to analyse the different hydrogen bonds formed between FTCA and 2-AP in crystal structure of (FTCA)^−^(2-AP)^+^ salt. The topological parameters of different interactions present in salt are summarised in Table S4.† FTCA interacts with 2-AP *via* proton transferred hydrogen bond (C23–O3⋯H26–N^+^25) and intermolecular hydrogen bond (C23–O5⋯H30–N28) such that the electron density and their topological characteristics are significantly different. The high negative values of ∇^2^*ρ*_(*r*)_ for N^+^25–H26 (−3.950560) and N28–H30 (−3.713842) indicate the strong covalent bond between N and H whereas the high positive values of ∇^2^*ρ*_(*r*)_ for O^−^3–H26 (+0.171243) and O5–H30 (+0.119708) indicate electrostatic and weak interactions between O and H. In generalisation to topological features, the high negative value of ∇^2^(*r*) corresponds high (*r*), lower bond stretch (BPL-GBL I), lower kinetic energy (*K*) and lower ellipticity (*ε*).^66^

**Fig. 6:**
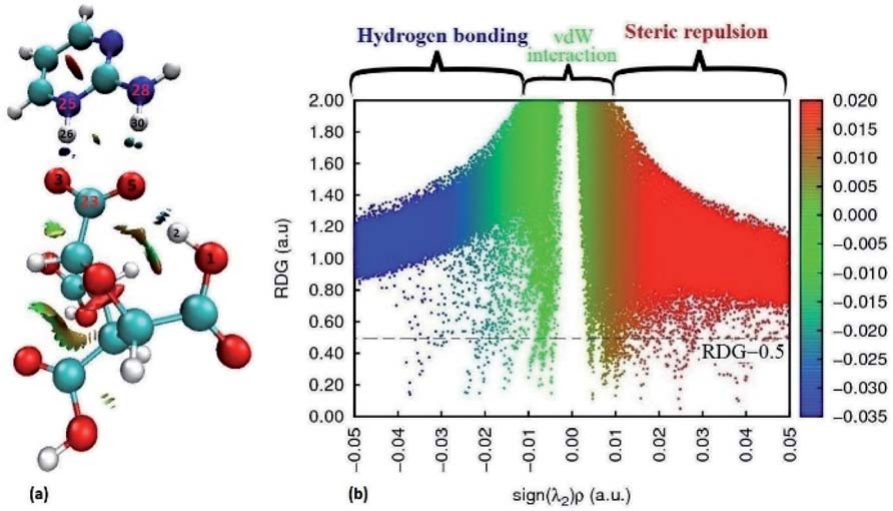
Standard NCI index representations (a) isosurface maps and (b) RDG scatter plots at B3LYP-D3/6-311G level. The RDG cut-off is sign(*λ*_2_)*ρ* = 0.5 a.u, and the colour scale is – 0.035 to 0.02 a.u. (blue, green and red surfaces indicate attractive, van der Waals, and repulsive interactions, respectively).

Furthermore, the reduced density gradient (RDG) analysis have been done to investigate the repulsive and non-covalent interactions (NCIs) in real space based on molecular geometry information and graphical visualization.^67,68^ In colour coding, red stands for strong repulsive, blue for strong attractive, green for van der waals (VDW) whereas mixed colour for mixed interactions. The 3D isosurfaces and scatter diagram for (FTCA)^−^(2-AP)^+^ salt are shown in Fig. 6. In the NCI-RDG isosurface illustration, the blue spots between the hydrogen atom (H26) of 2-AP and oxygen atom (O^−^3) of FTCA occurs due to formation of cation–anion interaction *via* proton transferred hydrogen bond (C23–O^−^3⋯H26–N^+^25). The blue dots between H30 of 2-AP and O5 of FTCA appears due to formation of inter molecular hydrogen bonds, whereas the blue dots between H2 and O5 of FTCA appears due to intramolecular hydrogen bonding. In addition, the red dots appearing in the centre of aromatic rings indicates steric repulsion effects. The RDG scatter plot determines the strength of the weak interaction based on the peaks at low and high densities. The value of RDG *versus* sign (*λ*_2_)*ρ* is calculated with the contour value set to 0.5 a.u. and the value of isosurfaces from −0.05 to 0.05 a.u (deep blue to deep red). From Fig. 6(b), positive sign(*λ*_2_)*ρ* stands for steric effects, negative for hydrogen bonding interactions, and the van der Waals (VDW) effects are shown by values near zero.

Natural Bond Orbital (NBO) analysis is an important program to understand the occurrence of different interactions *viz.* electronic conjugation, conjugative interactions, and hydrogen bond interactions. All possible donor–acceptor interactions have been analysed using the theory of second order perturbations of the off-diagonal Fock matrix element between “filled” Lewis-type NBOs (donor) and “empty” non-Lewis NBOs (acceptor).^69,70^ The intensity of interaction between NBO donors and NBO acceptors is well expressed by stabilization energy E^(2)^ (kcal mol^−1^) (Table 4). The NBO parameters associated with the hydrogen bond interactions involving LP (Lone pair) → σ* (anti-bonding) transition between 2-AP and FTCA have been studied by taking into account only hyperconjugative interactions with high stabilization energy. Hyperconjugative interaction between FTCA and 2-AP due to LP(O3) → σ*(N25–H26) and LP (O5) → σ* (N28–H30) stabilizes the salt with stabilization energy 8.98 and 4.70 kcal mol^−1^ respectively. Thus, the donation of electrons from O3 and O5 LP orbitals to σ* orbitals contribute to the formation of the stabilized salt structure. The another charge transfer within the FTCA moiety occurs due to LP(O5) → σ*(O1–H2) with stabilization energy 8.29 kcal mol^−1^ confirming the presence of intramolecular hydrogen bond in (FTCA)^−^(2-AP)^+^ salt.

**Table 4:** NBO analysis of the hydrogen bonds that influence the system, based on the optimized geometry SP-DFT/B3LYP/6-311G(d,p) level theory (donor–acceptor natural bond orbital interactions with second-order perturbation stabilization energy *E*^(2)^ (kcal mol^−1^))^a^

Bond	Orbital interaction			*E* ^(2)^ kcal mol^−1^
	Donor NBO		Acceptor NBO	
N25–H26⋯O3	LP(1)O3	→	σ*(1)N25–H26	3.09
	LP(2)O3	→	σ*(1)N25–H26	8.98
N28–H30⋯O5	LP(1)O5	→	σ*(1)N28–H30	1.72
	LP(2)O5	→	σ*(1)N28–H30	4.70
	LP(3)O5	→	σ*(1)N28–H30	0.64
O1–H2⋯O5 (intramolecular)	LP(1)O5	→	σ*(1)O1–H2	3.81
	LP(2)O5	→	σ*(1)O1–H2	8.29

a LP = lone pair electron; σ* = anti-bonding orbitals.

### 3.4. Hirshfeld surface analysis, interaction energy and energy frameworks

**Fig. 7:**
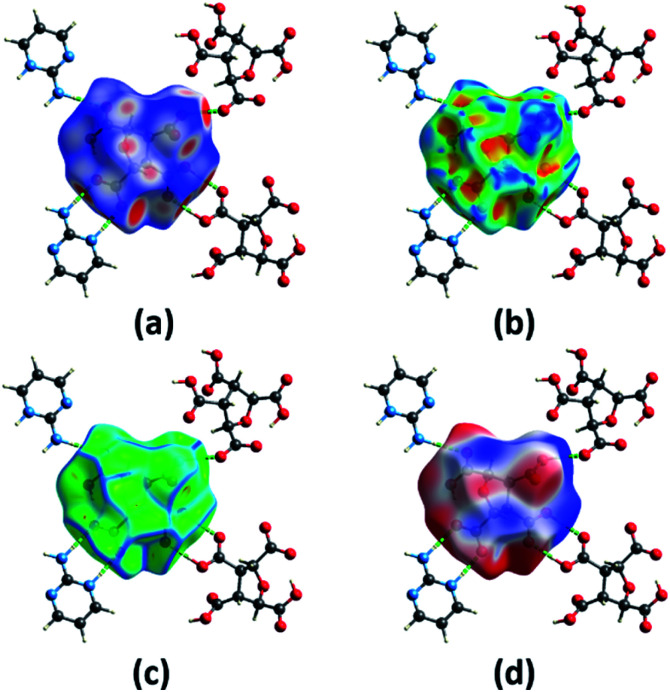
A view of Hirshfeld surface for (FTCA)^−^(2-AP)^+^ salt mapped over (a) *d*_norm_ (b) shape-index (c) curvedness (d) electrostatic potential.

The different computation studies discussed in Sections 3.2 and 3.3 were done on the optimized structure of (FTCA)^−^(2-AP)^+^ salt such that only asymmetric unit is taken. The Hirshfeld surfaces (HS) analysis was done for quantitative and qualitative analysis of robust synthons and for gaining a better understanding of the overall packing pattern of the salt. The HS (mapped over different parameters) were generated on FTCA moiety of the salt for understanding the effect of neighbouring groups in salt formation. Fig. 7(a) shows HS mapped over *d*_norm_ such that the red, white, and blue colour conventions recognized the interatomic contacts were short, at van der Waals separation and longer interatomic distances, respectively. The bright red spots on the surface indicated the presence of O–H⋯O & N–H⋯O contacts and facilitated the formation of homodimer and heterodimer synthon. Further chemical insight into molecular packing was done by mapping the HS over two useful measures of curvature, namely shape index and curvedness. These two properties were employed to investigate the weak interactions in salt and overall packing of crystal. The absence of red and blue triangles on shape-indexed mapped surface and absence of flat region on the curvedness mapped surface indicated the absence of π⋯π stacking in salt (Fig. 7(b) and (c)). Another HS were mapped over the calculated electrostatic potential (ESP) on the B3LYP/6-31G(d,p) model (Fig. 7(d)). The donor atoms had positive electrostatic potential shown by blue regions, while acceptor atoms had negative potential shown by red regions. The ESP calculated by DFT studies (Fig. 4(a), Section 3.2) predicted the donor and acceptor sites in FTCA and matched well with ESP of salt calculated on HS.

**Fig. 8:**
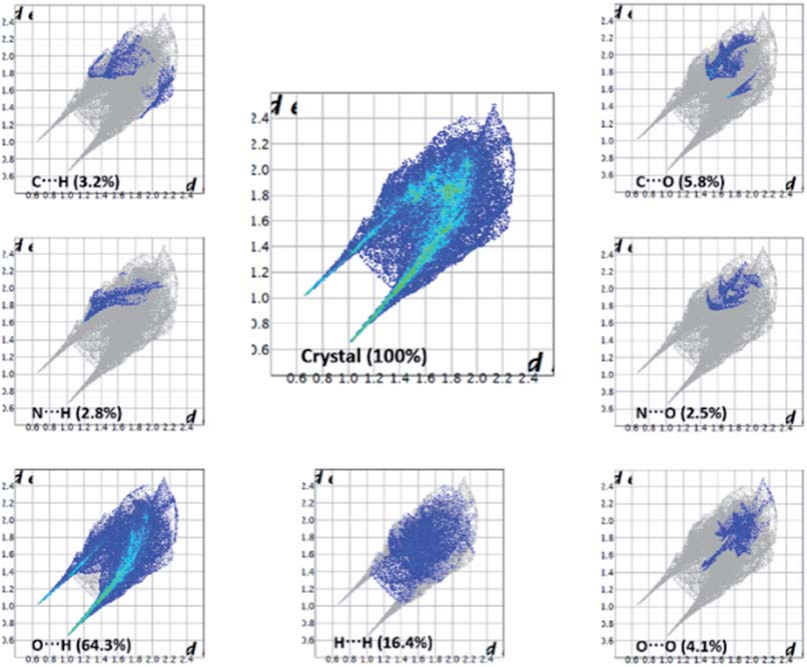
2-D fingerprint plots for (FTCA)^−^(2-AP)^+^ salt.

The important quantitative information of the intermolecular interactions in (FTCA)^−^(2-AP)^+^ salt was obtained by plotting two-dimensional (2D) fingerprint plots (overall as well as delineated) shown in Fig. 8. The overall fingerprint plot encompassed all intermolecular interactions, whereas delineated (or decomposed) fingerprint plots depicted specific interactions. The forceps-like tips in 2D fingerprint plots appeared due to significant contribution of O⋯H/H⋯O interactions. The H⋯H significant contribution appeared as asymmetric points spread over a large area as broad peaks. Only those closed contacts were considered in which the percent contribution was above 1%. The strong contacts (O⋯H/H⋯O) contributed 64.3% of total interaction to the HS. The weakest contacts (H⋯H) also contributed a significant contribution 16.4% of the total interaction to HS (Fig. 8).

**Fig. 9:**
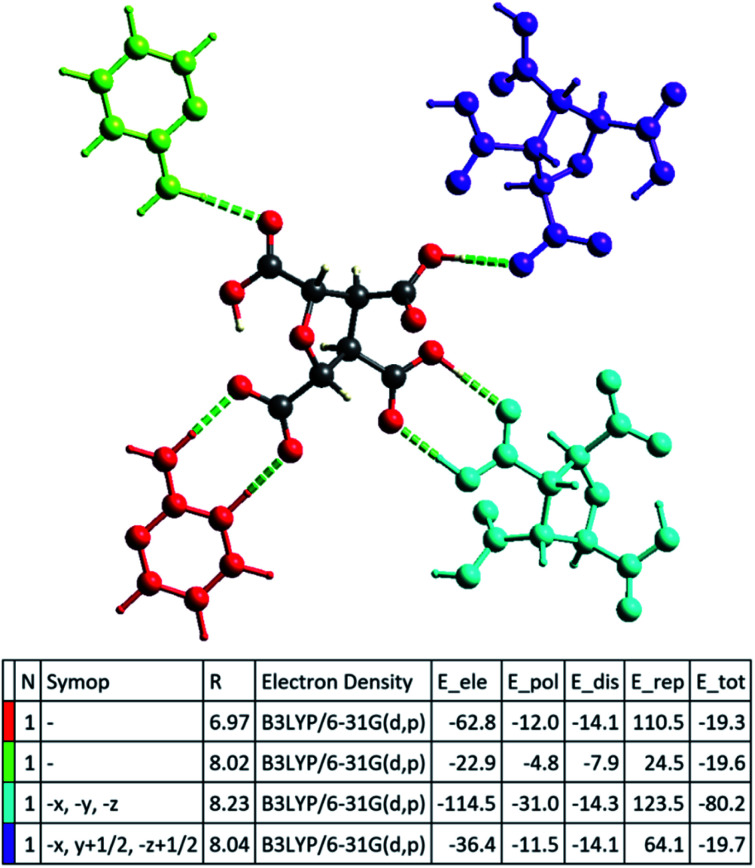
Colour-coded total interaction energies calculated for (FTCA)^−^(2-AP)^+^ salt. The individual electrostatic, polarization, dispersion and exchange-repulsion energies with scale factors of 1.057, 0.740, 0.871 and 0.618, respectively are also shown.

The successful calculation of specific, as well as total, interaction energy in a colour-coded molecular cluster, was done for (FTCA)^−^(2-AP)^+^ salt (Fig. 9). The total intermolecular interaction energy was −80.2 kJ mol^−1^ for two-point homosynthon while −19.7 kJ mol^−1^ for one-point homosynthon. However, the total intermolecular interaction energy for heterosynthons were comparatively low; −19.6 kJ mol^−1^ for one-point heterosynthon while −19.3 kJ mol^−1^ for proton transferred two-point heterosynthon. All the total intermolecular interaction energies were comparable to strong hydrogen bonding. As stated earlier, HS analysis mapped over *d*_norm_ is used to identify the close contacts such that the strength of these contacts may be estimated qualitatively by the intensity of red spots. This new feature (interaction energy calculations) of Crystal Explorer helps to quantify the strength of contacts that are correlated with the results obtained by HS analysis. Apart from energy data, lattice energy of a crystal can also be calculated from the generated table, such as number of pair(s) of interacting molecules with respect to the reference molecule (N), centroid-to-centroid distance between the reference molecule and interacting molecules (R), and existence of rotational symmetry operations with respect to the reference molecule (Symop).

**Fig. 10:**
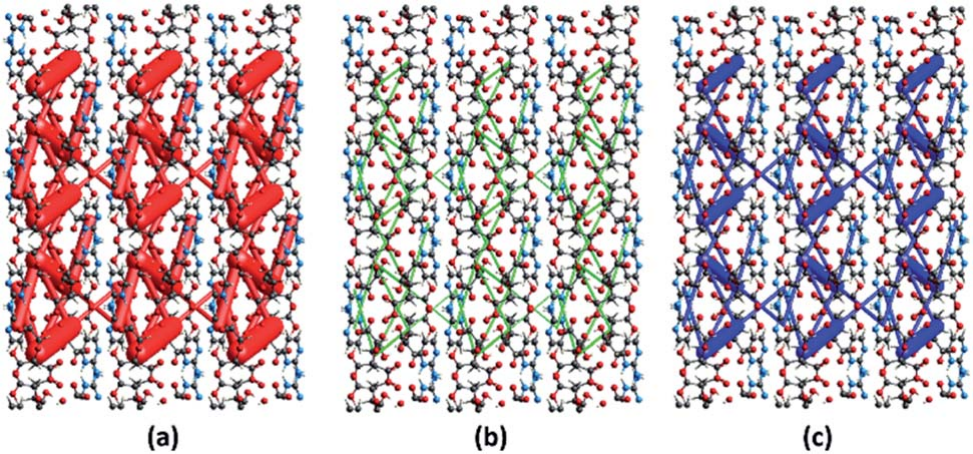
Energy frameworks calculated for (FTCA)^−^(2-AP)^+^ salt viewed along bc plane, showing (a) electrostatic potential force, (b) dispersion force, (c) total energy diagrams. The cylindrical radii are proportional to the relative strength of the corresponding energies and they were adjusted to the same scale factor of 100 with a cut-off value of 3 kJ mol^−1^ within 3 × 3 × 3 unit cells.

The energy frameworks were also generated for a cluster of 3 × 3 × 3 unit cells using same quantum level of theory as mentioned for interaction energy model. The study is helpful for better understanding of the topology of overall interaction energies between the constituents of (FTCA)^−^(2-AP)^+^ crystal (Fig. 10). The crystal was significantly governed by electrostatic force owing to the strong O⋯H/H⋯O interactions that resulted in a zig-zag shape energy topology across the bc plane. Though less significant, an essential dispersion contribution was also observed that arose due to π⋯π interactions spanning all the aromatic rings. Overall, we conclude that these interacting forces directed the assembly of the molecules in the salt.

## 4. Conclusions

The current study, highlights the increase the predictability of the elusive range of Δp*K*_a_ for acid-aminopyrimidine (specifically 2-AP) robust synthon. The in-depth analyses of CSD data and Δp*K*_a_ values, a generalization has been made such that if two or more substituting groups, having negative inductive effect (−*I* effect), are present on an aromatic acid ring, then the proton transfer will occur from aromatic acid to 2-AP. The plausible reason behind the generalization is the increase in stability of carboxylate ion by –*I* groups. Following the proposed generalization a crystallization experiment was set up on an aromatic tetracarboxylic acid (FTCA) and 2-AP to verify the predicted proton transfer and the formation of a novel (FTCA)^−^(2-AP)^+^ salt. Earlier, the authors of the same lab concluded that LHT is robust over HT supramolecular synthon, and in the present case a rare, CH-LHT synthon is formed. The homodimer interactions present here are rare R^2^_2_(6) C–H⋯N interactions mimics R^2^_2_(8) N–H⋯N homodimer interactions of previous cases.^33,34^

DFT calculations of (FTCA)^−^(2-AP)^+^ salt and its constituent molecules were carried out to understand the nature of molecules by applying (DFT/B3LYP-D3) method with the 6-311G(d,p) basis set in the gas and ethanol. The obtained geometrical parameters (bond lengths, angles, and dihedral angles) at IEF-PCM-B3LYP-D3/6-311G(d,p) were compared with SCXRD data of (FTCA)^−^(2-AP)^+^ salt such that most parameters of atoms were in good agreement with experimental data. Moreover, the optimized salt structure using DFT approach was found to be most stable among other applied computational methods *viz. ab initio* (IEF-PCM-MP2/6-311G(d,p) and IEF-PCM-HF/6-311G(d,p)) and semi-empirical (IEF-PCM-PM6) with the highest negative SCF energy, *i.e.*, −820087.24 kcal mol^−1^. DFT calculations were also done on a hypothetical cocrystal structure using the same basis set as in salt optimization to calculate and compare the binding energy (B.E.) of salt and cocrystal. In close observation, it was found that the higher B.E. favoured salt formation (B.E. = −188.78 kJ mol^−1^) over cocrystal (B.E. = −68.58 kJ mol^−1^). Energy gap, Δ*E*_g_ (calculated by using FMOs), of (FTCA)^−^(2-AP)^+^ salt decreased significantly compared to its constituents (FTCA and 2-AP), thereby lower kinetic stability and high chemical reactivity of salt in the gas phase but the salt was kinetic stabilized in the solution phase. MESP diagrams of salt and its constituents displayed reactive sites on the surface responsible for atoms' interactions in chemical species. Non-covalent interaction of isolated (FTCA)^−^(2-AP)^+^ were investigated using QTAIM, RDG, and NBO analyses. The study of spikes on the RDG surface classified interactions involved within salts such as van der Waals (vdW), hydrogen bond interactions, and stearic effect. Second-order perturbation theory was applied under NBO approach and showed that the donor–acceptor interactions LP(O3) → σ*(N25–H26) and LP (O5) → σ* (N28–H30) were responsible for the weak interactions in the salt. Hirshfeld surface analyses concluded that the major interactions present in salt were strong O⋯H/H⋯O interactions (contributed 64.3%) whereas weak H⋯H interactions (contributed 16.4%) also had a significant contribution. The interaction energy calculations in 3-D space were also found in strong hydrogen bonding region, which were in good agreement with NCI-RDG plots. Total energy frameworks concluded a zig-zag topology in which the electrostatic force played a dominant role in stabilizing the overall crystal structure.

## Conflicts of interest

There are no conflicts to declare.

## Supplementary Material

RA-011-D1RA01714D-s001

RA-011-D1RA01714D-s002
