# Supplementary material for: In acid-aminopyrimidine continuum: experimental and computational studies of furan tetracarboxylate-2-aminopyrimidinium salt
Source: RSC Adv. 2021 Jun 17;11(35):21463–74. doi: 10.1039/d1ra01714d (PMC9034213; doi:10.1039/d1ra01714d)
Supplement: RA-011-D1RA01714D-s001 [file RA-011-D1RA01714D-s001.pdf]

Electronic Supplementary Material (ESI) for RSC Advances.
This journal is © The Royal Society of Chemistry 2021

Electronic Supplementary Information

In acid-aminopyrimidine continuum: Experimental and computational studies of Furan tetracarboxylate-2-aminopyrimidinium salt

Utsav Garg[a], Yasser Azim*[a], Mahboob Alam*[b]

[a]Department of Applied Chemistry, Zakir Husain College of Engineering & Technology, Faculty of Engineering & Technology, Aligarh Muslim University, Aligarh, 202002, Uttar Pradesh, India.

[b]Division of Chemistry & Biotechnology, Dongguk University, 123 Dongdae-ro, Gyeongju, Republic of Korea

* Corresponding author; e-mail: yasser.azim@gmail.com (YA), mahboobchem@gmail.com (MA)

Content

Table S1 Crystallographic data and experimental details of $(FTCA)^{-}(2\text{-}AP)^{+}$ salt

Table S2 Selected hydrogen-bond geometry of $(FTCA)^{-}(2\text{-}AP)^{+}$ salt

Table S3 Comparison of selected experimental structural parameters (bond lengths, bond angles, and dihedral angles) with optimized structural parameters of $(FTCA)^{-}(2\text{-}AP)^{+}$ salt.

Table S4 Calculated quantum chemical parameters (in eV) of FTCA, 2-AP and $(FTCA)^{-}(2\text{-}AP)^{+}$ salt using DFT/B3LYP-D3 method in the gas phase.

Table S5 Characteristics bond critical point (BCP) of $(FTCA)^{-}(2\text{-}AP)^{+}$salt

Fig. S1 Comparison of binding energies (B.E.) of (a) optimized salt structure of $(FTCA)^{-}(2\text{-}AP)^{+}$, and (b) optimized hypothetical structure of cocrystal between FTCA and 2-AP to demonstrate salt formation over the cocrystal formation.

Fig. S2 Molecular orbital surfaces and energies for the HOMO and LUMO of $(FTCA)^{-}(2\text{-}AP)^{+}$ including energies gap using same level theory in gas phase.

Fig. S3 Molecular orbital surfaces and energies for the HOMO and LUMO of (a) 2-aminopyrimidine (2-AP) and (b) Furantetracarboxylic acid (FTCA) including energies gap using same level theory in gas phase.

Fig. S4 Molecular orbital surfaces and energies for the HOMO and LUMO of (a) 2-aminopyrimidine (2-AP) and (b) Furantetracarboxylic acid (FTCA) including energies gap using same level theory in the solution phase.

Fig. S5 Molecular electrostatic potential (MESP) formed by mapping of the total density over electrostatic potential in gas phase for (a) 2-aminopyrimidine (2-AP) and (b) Furantetracarboxylic acid (FTCA) using same level theory in ethanol.

Table S1 Crystallographic data and experimental details of $(FTCA)^{-}(2\text{-}AP)^{+}$ salt

Parameters	$(FTCA)^{-}(2\text{-}AP)^{+}$
CCDC no.	2057691
Empirical Formula	$C_{12}H_{13}N_3O_9$
Formula weight	343.25
Crystal size/mm	0.20 × 0.15 × 0.09
Crystal system	Monoclinic
Unit cell dimensions	a = 10.8226 (9) Å<br>b = 13.7338 (8) Å<br>c = 9.9166 (9) Å<br>$\alpha = \gamma = 90^0$<br>$\beta = 106.482^0$
Space group	$P2_1/c$
Z	4
Temperature (K)	293
Wavelength (Å)	1.54184 Å
Volume ($Å^3$)	1413.4 (2)
Density (g cm^{-3})	1.613
μ/mm^{-1}	1.229
F(000)	712
ϑ range	3.7^0 - 66.7^0
Max/min. indices *h,k,l*	-12 ≤ h ≤ 12,-12 ≤ k ≤ 16, -11 ≤ l ≤ 11
Measured reflections	4424
Independent reflections	2459 (R_{int} = 0.032)
Observed reflections [$I \geq 2\sigma(I)$]	1955
Goodness-of-fit on F^2	1.09
Radiation type	CuK_{α}
h, k, l max	12,16,11
Final R indices<br>[$I \geq 2\sigma(I)$]	$R1$ = 0.045<br>$wR2$ = 0.123

Table S2 Selected hydrogen-bond geometry of $(FTCA)^{-}(2\text{-}AP)^{+}$ salt

D—H···A	D—H	H···A	D···A	D—H···A
O4—H4···O2	0.82	1.82	2.605 (2)	160
O6—H6···O1i	0.82	1.85	2.662 (2)	173
N1—H1···O1	0.86	1.86	2.711 (2)	170
N2—H2B···O2	0.86	1.99	2.853 (2)	178
O8—H8···O9ii	1.02 (7)	1.66 (7)	2.676 (3)	176 (6)

Symmetry codes: (i) -*x*+1, *y*+1/2, -*z*+1/2; (ii) -*x*+1, -*y*+1, -*z*+1

Table S3 Comparison of selected experimental structural parameters (bond lengths, bond angles, and dihedral angles) with optimized structural parameters of $(FTCA)^{-}(2\text{-}AP)^{+}$ salt.

Bonds (Å)	DFT*	Expt	Angles (°)	DFT*	Expt	Dihedral angles (°)	DFT*	Expt
O1—H2	0.98	0.820	H2—O1—C18	112.71	109.47	H2—O1—C18—O11	173.81	174.47
O1—C18	1.33	1.302	C23—O3—H26	112.03	123.45	H2—O1—C18—C21	-5.379	-7.59
O3—C23	1.29	1.253	C19—O4—C21	111.31	111.96	C21—O4—C19—C14	-4.62	-0.92
O3—H26	1.569	1.860	C23—O5—H30	123.48	109.99	H30—O5—C23—O3	0.19	23.78
O4—C19	1.432	1.425	H7—O6—C16	107.35	109.48	H30—O5—C23—C19	179.18	-158.3
O4—C21	1.427	1.413	H10—C9—C14	113.69	111.33	C23—O5—N28—H29	179.60	159.62
O5—C23	1.23	1.250	H10—C9—C16	109.15	111.34	C23—O5—N28—C31	-4.99	-22.02
O5—H30	1.914	1.994	H10—C9—C21	110.81	111.30	H7—O6—C16—O8	-0.56	12.64
O6—C16	1.346	1.314	C14—C9—C16	112.10	112.17	H15—C14—C19—C23	30.40	23.10
O8—C16	1.204	1.201	C14—C9—C21	100.64	101.55	C17—C14—C19—O4	153.95	145.04
C9—H10	1.088	0.980	C16—C9—C21	110.18	108.74	C17—C14—C19—C23	-88.51	-96.20
C9—C14	1.539	1.549	C17—O12—H24	107.01	112.29	O1—C18—C21—O4	-9.11	-13.86
C9—C16	1.517	1.509	C9—C14—H15	107.26	108.46	O4—C19—C23—O3	-142.18	-152.71
C9—C21	1.542	1.542	C9—C14—C17	116.90	115.95	O4—C19—C23—O5	38.72	29.21
O11—C18	1.203	1.210	H15—C14—C17	106.37	108.45	H26—N25—C31—N27	-179.03	-179.03
N25—H26	1.073	0.860	C17—C14—C19	114.01	111.46	H26—N25—C31—N28	0.80	0.69
N25—C31	1.357	1.347	O1—C18—O11	123.07	121.33	H30—N28—C31—N25	2.44	0.26
N25—C32	1.336	1.350	H26—H25—C31	125.51	119.51	H30—N28—C31—N27	-177.70	179.98
N27—C31	1.351	1.341	H26—N25—C32	117.23	119.51	C34—N27—C31—N25	-0.14	-0.28
N27—C34	1.325	1.324	C31—N25—C32	117.23	120.97	C21—C9—C16—O8	103.31	59.95
N28—H29	1.005	0.860	C31—N27—C34	116.56	117.01	H26—N25—N28—H30	2.75	0.83
N28—H30	1.016	0.860	H29—C28—H30	121.17	120.0	H30—O5—O3—H26	3.24	12.57
N28—C31	1.342	1.321	H29—C28—C31	117.87	120.0	O3—H26—N25—C31	-176.66	68.04
H30—O5	1.825	1.819	N25—C31—N28	117.83	119.36	O5—H30—N28—H29	-173.27	-55.48
O5—H2	1.825	1.819	N25—C31—N27	124.47	121.60	O5—H2—O10—C18	-116.44	-144.42

Note: Numbering scheme is taken from Figure 2(b), Expt. refers to experimental

Table S4 Calculated quantum chemical parameters (in eV) of FTCA, 2-AP and $(FTCA)^{-}(2\text{-}AP)^{+}$ salt using DFT/B3LYP-D3 method in the gas phase.

Parameters	FTCA	2-AP	$(FTCA)^{-}(2\text{-}AP)^{+}$ salt
E_{LUMO}	-1.35	-0.06	-3.70
E_{HOMO}	-7.95	-6.98	-4.09
E_g (E_{LUMO} - E_{HOMO})	6.6	6.92	0.39
Minimum SCF energy (kcal/mol)	-619368.66	-200668.97	-820069.976
Dipole Moment (Debye)	1.73	0.15	4.81
Ionization Potential (I)	7.95	6.98	4.09
Electron Affinity (A)	1.35	0.06	3.70
Chemical Hardness (η)	3.3	3.46	0.195
Chemical Softness (S)	0.151	0.144	2.564
Electronegativity (χ)	4.65	3.52	3.895
Electronic Chemical potential (μ)	-4.65	-3.52	-3.895
Electrophilicity index (ω)	3.264	1.784	38.898

Table S5 Characteristics bond critical point (BCP) of $(FTCA)^{-}(2\text{-}AP)^{+}$ salt

BCP	Atoms	$\rho_{(r)}$	$\nabla^2\rho_{(r)}$	ε	V	G	K	H	BPL - GBL_I
1	O1—H2	0.549581	-5.129800	0.018385	-1.484419	0.100984	1.383435	-1.383435	0.001810
2	O5—C23	0.365135	-0.653109	0.156692	-0.984729	0.410726	0.574003	-0.574003	0.001366
3	C19—C23	0.252109	-0.635125	0.043472	-0.231494	0.036356	0.195138	-0.195138	0.000086
4	H2—O5	0.027685	+0.18911	0.102377	-0.03992	0.043635	-0.003643	0.003643	0.006070
5	O6—C16	0.316095	-0.619221	0.133089	-0.783871	0.314533	0.469338	-0.469338	0.002188
6	O6—H7	0.550657	-4.840280	0.018799	-1.420702	0.105316	1.315386	-1.315386	0.001515
7	O4—C19	0.252977	-0.543028	0.045022	-0.533770	0.199006	0.334763	-0.334763	0.001151
8	O8—C16	0.405874	+0.03124	0.295447	-1332339	0.670075	0.662264	-0.662264	0.000250
9	C9—C16	0.256648	-0.639280	0.026637	-0.252089	0.046135	0.205955	-0.205955	0.000265
10	C14—C19	0.237437	-0.520530	0.038759	-0.222648	0.046258	0.176390	-0.176390	0.000695
11	C9—C21	0.243473	-0.546431	0.024972	-0.233632	0.048512	0.185120	-0.185120	0.000645
12	C9—C14	0.238354	-0.509832	0.008899	-0.228220	0.050381	0.177839	-0.177839	0.000508
13	C9—H10	0.364904	-1.540204	0.009138	-0.519414	0.067182	0.452233	-0.452233	0.000008
14	O1—C18	0.329425	-0.713756	0.101839	-0.82487	0.323219	0.501658	-0.501658	0.001494
15	C18—C21	0.250848	-0.630828	0.053287	-0.234775	0.038534	0.196241	-0.196241	0.000438
16	O3—O13	0.008336	+0.03224	0.160018	-0.007285	0.007674	-0.000388	0.000388	0.010721
17	C14—C17	0.254336	-0.631323	0.029741	-0.249212	0.045961	0.203521	-0.203521	0.000842
18	O13—C17	0.377969	-0.085446	0.348124	-1.177950	0.578294	0.599656	-0.599656	0.001469
19	C14—H15	0.366838	-1.547063	0.006634	-0.526473	0.069853	0.456619	-0.456619	0.000006
20	O12—H24	0.317914	-2.091880	0.021199	-0.609071	0.043051	0.566021	-0.566021	0.000900
21	O12—C17	0.344599	-0.213600	0.113631	-0.992232	0.469416	0.522816	-0.522816	0.001806
22	O11—C18	0.401105	-0.067132	0.285056	-1.286232	0.634724	0.651507	-0.651507	0.000397
23	C19—H20	0.368334	-1.595821	0.030530	-0.517054	0.059050	0.458005	-0.458005	0.000079
24	O4—C21	0.263418	-0.619331	0.052637	-0.549039	0.197103	0.351936	-0.351936	0.001353
25	C21—H22	0.370871	-1.611238	0.027735	-0.523626	0.060408	0.463218	-0.463218	0.000016
26	O3—C23	0.361260	-0.306431	0.176685	-1.052879	0.488136	0.564744	-0.564744	0.001680
27	O3—H26	0.024620	+0.17124	0.037392	-0.032682	0.037746	-0.005064	0.005064	0.006660
28	N25—C32	0.322182	-1.109153	0.041830	-0.595952	0.159332	0.436620	-0.436620	0.000079
29	N25—H26	0.506334	-3.950560	0.024286	-1.143799	0.078080	1.065720	-1.065720	0.000010
30	C34—H35	0.407415	-1.944373	0.005242	-0.640796	0.077351	0.563445	-0.563445	0.000211
31	N25—C31	0.333865	-1.049168	0.288082	-0.588566	0.163137	0.425429	-0.425429	0.000071
32	O5—H30	0.019409	+0.11970	0.109158	-0.021829	0.025878	-0.004049	0.004049	0.001227
33	N28—H30	0.498197	-3.713842	0.056692	-1.117280	0.094409	1.022870	-1.022870	0.000042
34	N27—C31	0.351033	-1.344314	0.047119	-0.521122	0.092522	0.428600	-0.428600	0.003263
35	N27—C34	0.347400	-1.022200	0.322687	-0.741850	0.243150	0.498700	-0.498700	0.003961
36	N28—C31	0.355260	-1.374654	0.027833	-0.567744	0.112040	0.455704	-0.455704	0.000501
37	N28—H29	0.496764	-3.569117	0.063753	-1.093863	0.100792	0.993071	-0.993071	0.000119
38	C32—C36	0.327626	-0.879209	0.521160	-0.486805	0.133502	0.353304	-0.353304	0.000151
39	C36—H37	0.397027	-1.784121	0.041522	-0.643938	0.098954	0.544984	-0.544984	0.000003
40	C34—C36	0.309102	-0.980157	0.035285	-0.375861	0.065411	0.310450	-0.310450	0.000331
41	C32—H33	0.403424	-1.908625	0.009452	-0.629064	0.075954	0.553110	-0.553110	0.000129

Note: BCP = Bond Critical Point; $\rho_{(r)}$ = Electron Density; $\nabla^2\rho_{(r)}$ = Laplacian of $\rho_{(r)}$ = Trace of Hessian of $\nabla^2\rho_{(r)}$; ε = Ellipticity = (Hess $\rho_{(r)}$_EigVal(1)/ Hess $\rho_{(r)}$_EigVal(2)) – 1; V = potential energy density; G = kinetic energy density; K is hamiltonian form of kinetic energy density; H = total energy density; H = G + V is equivalent to -K, i.e., minus the Hamiltonian form of the electron kinetic energy; BPL – GBL_I = bond strain.

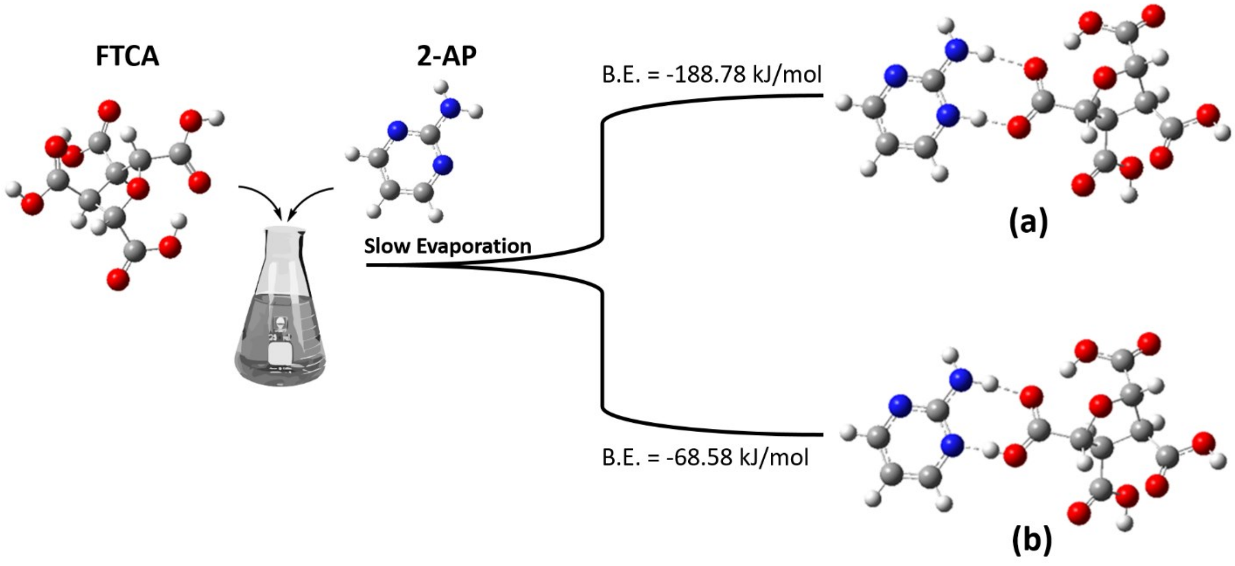

Fig. S1 Comparison of binding energies (B.E.) of (a) optimized salt structure of $(FTCA)^{-}(2\text{-}AP)^{+}$, and (b) optimized hypothetical structure of cocrystal between FTCA and 2-AP to demonstrate salt formation over the cocrystal formation.

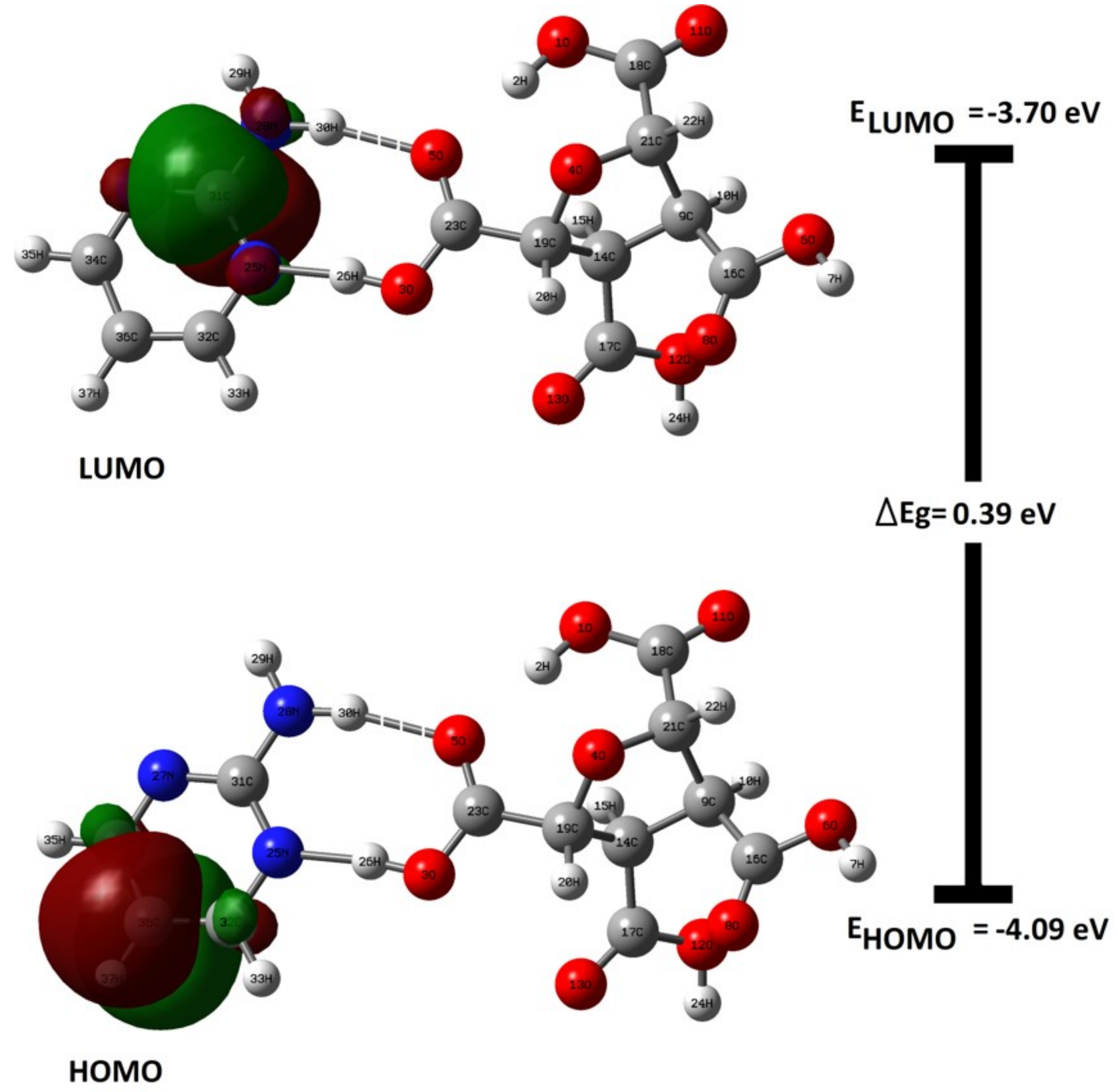

Fig. S2 Molecular orbital surfaces and energies for the HOMO and LUMO of $(FTCA)^{-}(2\text{-}AP)^{+}$ including energies gap using same level theory in gas phase.

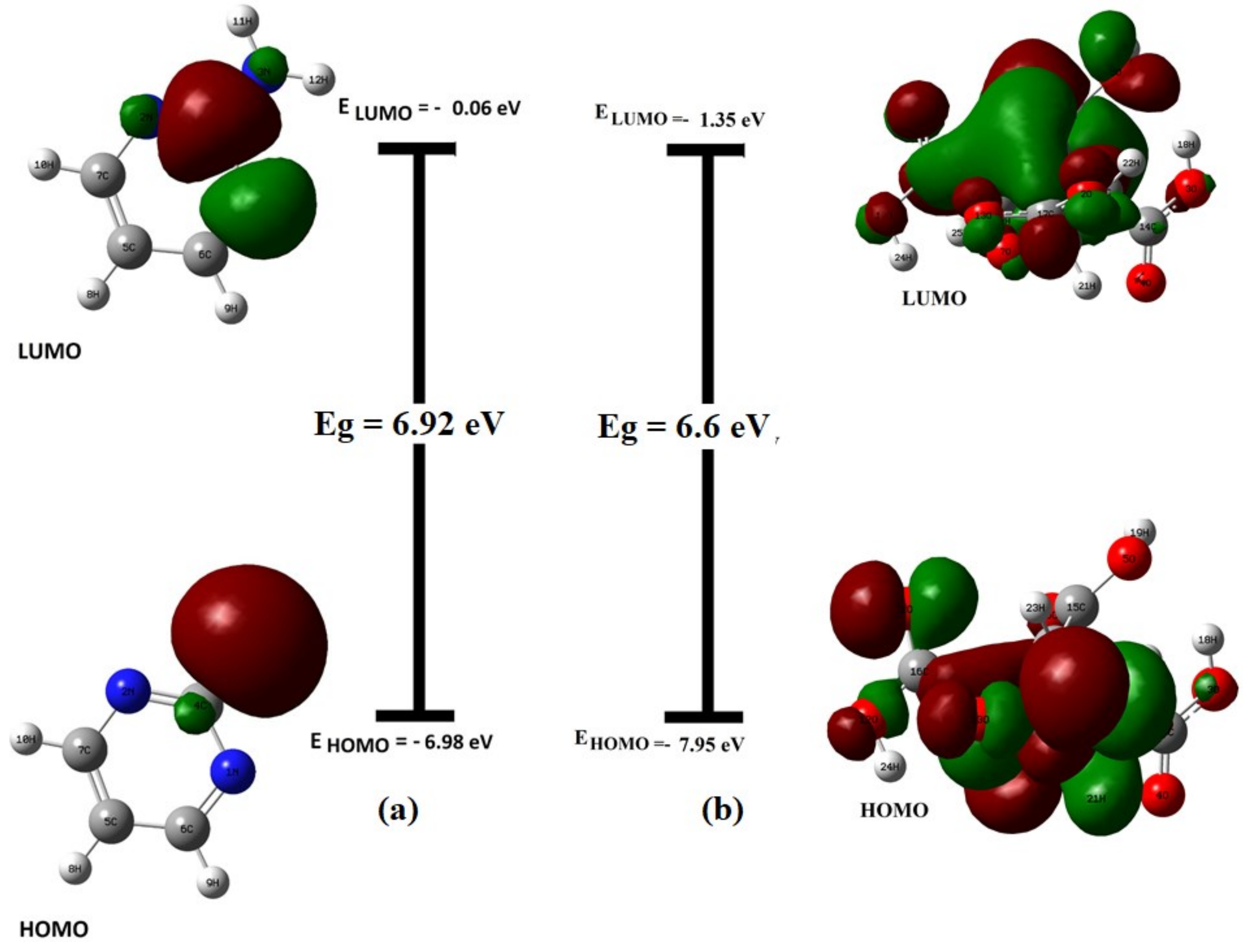

Fig. S3 Molecular orbital surfaces and energies for the HOMO and LUMO of (a) 2-aminopyrimidine (2-AP) and (b) Furantetracarboxylic acid (FTCA) including energies gap using same level theory in gas phase.

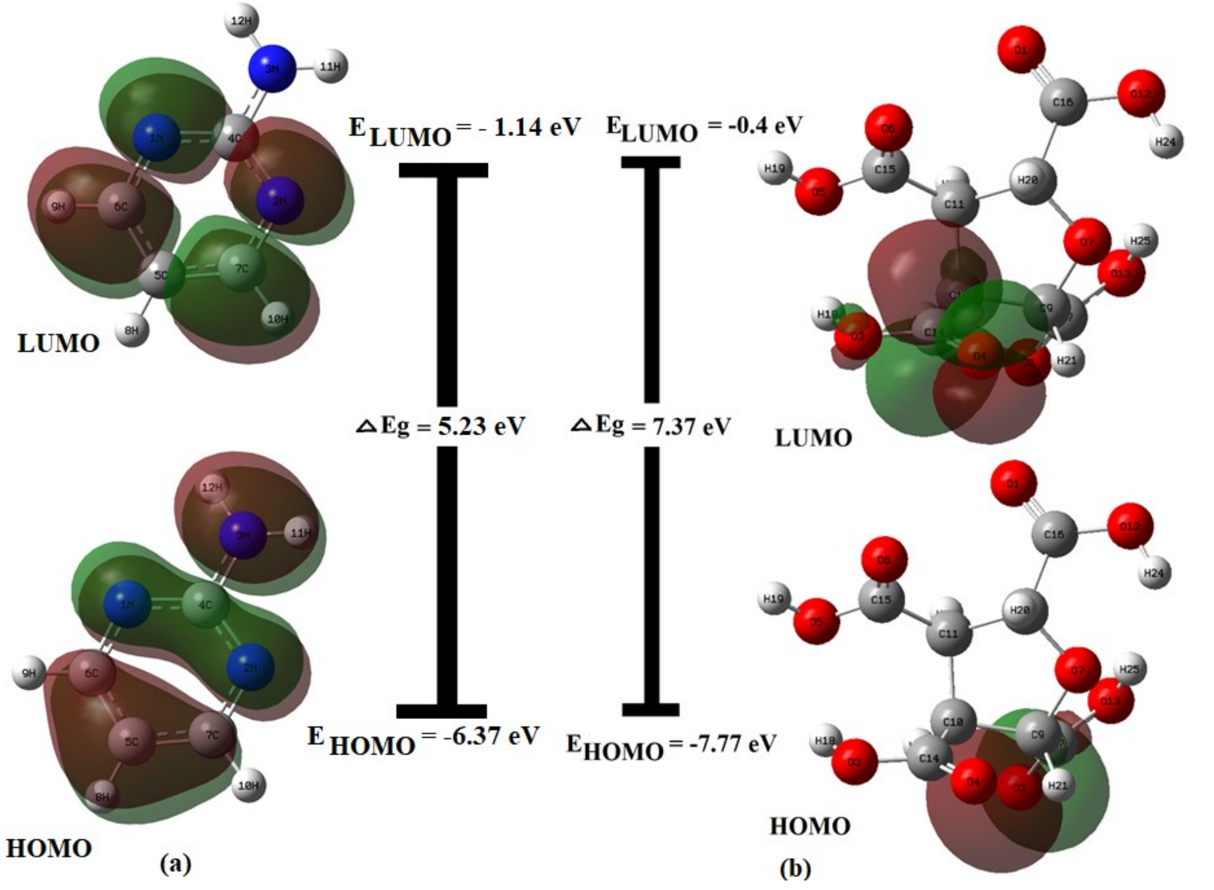

Fig. S4 Molecular orbital surfaces and energies for the HOMO and LUMO of (a) 2-aminopyrimidine (2-AP) and (b) Furantetracarboxylic acid (FTCA) including energies gap using same level theory in the solution phase.

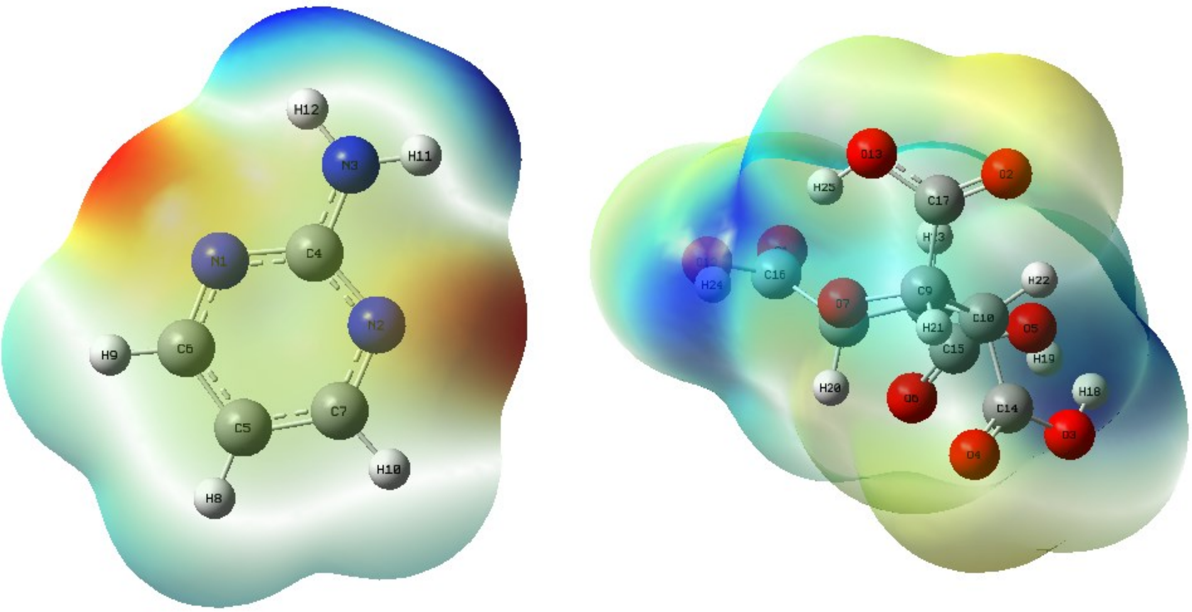

Fig. S5 Molecular electrostatic potential (MESP) formed by mapping of the total density over electrostatic potential in gas phase for (a) 2-aminopyrimidine (2-AP) and (b) Furantetracarboxylic acid (FTCA) using same level theory in ethanol.
